# Nanodrugs systems for therapy and diagnosis of esophageal cancer

**DOI:** 10.3389/fbioe.2023.1233476

**Published:** 2023-07-13

**Authors:** Lihan Zhang, Xing Li, Guangxing Yue, Lihao Guo, Yanhui Hu, Qingli Cui, Jia Wang, Jingwen Tang, Huaimin Liu

**Affiliations:** ^1^ Department of Integrated Chinese and Western Medicine, The Affiliated Cancer Hospital of Zhengzhou University and Henan Cancer Hospital, Zhengzhou, China; ^2^ Department of General Surgery, The Affiliated Cancer Hospital of Zhengzhou University and Henan Cancer Hospital, Zhengzhou, China; ^3^ Interdisciplinary Research Center of Smart Sensors, School of Advanced Materials and Nanotechnology, Xidian University, Xi’an, China

**Keywords:** nanomaterials, esophageal cancer, chemotherapy, radiotherapy, diagnosis

## Abstract

With the increasing incidence of esophageal cancer, its diagnosis and treatment have become one of the key issues in medical research today. However, the current diagnostic and treatment methods face many unresolved issues, such as low accuracy of early diagnosis, painful treatment process for patients, and high recurrence rate after recovery. Therefore, new methods for the diagnosis and treatment of esophageal cancer need to be further explored, and the rapid development of nanomaterials has brought new ideas for solving this problem. Nanomaterials used as drugs or drug delivery systems possess several advantages, such as high drug capacity, adjustably specific targeting capability, and stable structure, which endow nanomaterials great application potential in cancer therapy. However, even though the nanomaterials have been widely used in cancer therapy, there are still few reviews on their application in esophageal cancer, and systematical overview and analysis are deficient. Herein, we overviewed the application of nanodrug systems in therapy and diagnosis of esophageal cancer and summarized some representative case of their application in diagnosis, chemotherapy, targeted drug, radiotherapy, immunity, surgery and new therapeutic method of esophageal cancer. In addition, the nanomaterials used for therapy of esophageal cancer complications, esophageal stenosis or obstruction and oesophagitis, are also listed here. Finally, the challenge and the future of nanomaterials used in cancer therapy were discussed.

## 1 Introduction

Esophageal cancer ranks seventh globally in terms of its incidence in 2020, with 604,000 new cases reported, and it is the sixth leading cause of death, resulting in 544,000 fatalities. With approximately one in every 18 cancer-related deaths can be attributed to esophageal cancer (Sung et al., 2021), it poses a significant public health burden worldwide. The 5-year survival rate for esophageal cancer ranges from 10% to 30% in most countries, which is 29.7% in China and shows an improvement of 6%–10% since 2000. China is a high-incidence region for esophageal squamous cell carcinoma (ESCC). Currently, surgical intervention remains the primary treatment approach for resectable ESCC, but the efficacy of single-surgery is suboptimal, e.g., approximately 80% of patients experience recurrence or metastasis within 2 years post-operation. While radiotherapy and chemotherapy have shown promise in enhancing treatment outcomes, the efficacy of single-agent chemotherapy is 10%–20% far from satisfactory, and using combination regimens can only improve the efficacy to approximately 45%, accompanied by significant adverse effects. Consequently, there is an urgent need to explore novel and clinically effective treatment modalities to improve the therapeutic efficacy of patients diagnosed with esophageal cancer and inhibit recurrence.

**FIGURE 1:**
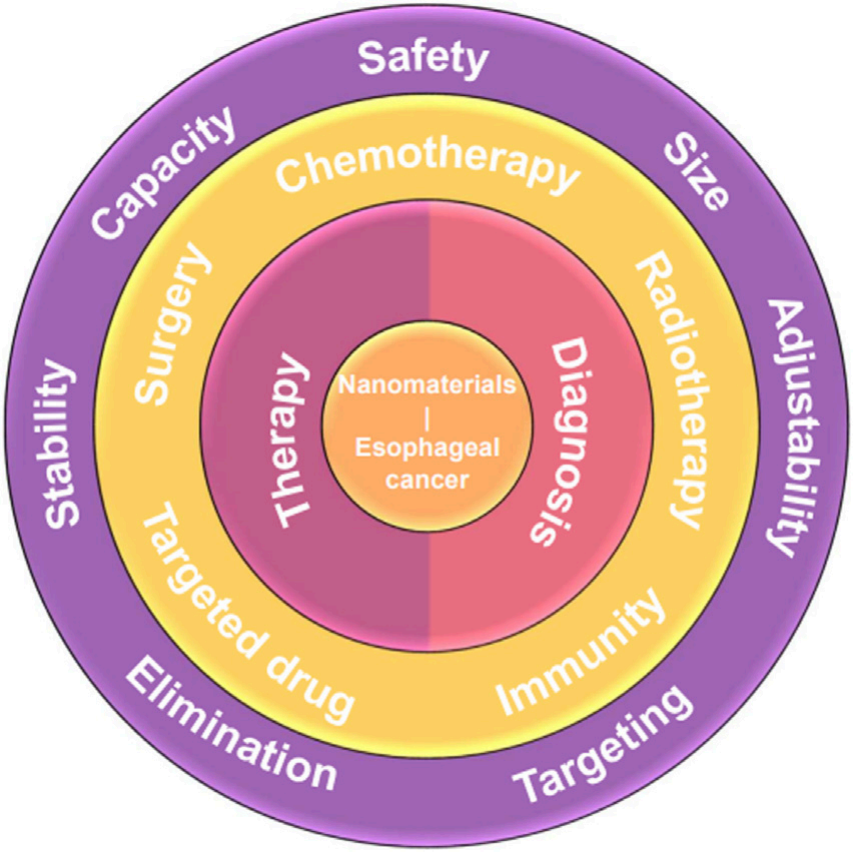
Schema of the main direction of nanomaterials for therapy and diagnosis of esophageal cancer. The nanomaterials for esophageal cancer (core) can be divided into two types, i.e., therapy and diagnosis (first ring). The nanomaterials for therapy of esophageal cancer can be specific to five aspects, i.e., chemotherapy, radiotherapy, targeted drugs, surgery, and immunity (second ring). The crucial criteria should be adhered in constructing nanocarriers for drug delivery systems.

Nanotechnology has paved a novel way in the medical field, and using nanocarriers as drug delivery systems has shown extraordinary potential to address many clinical difficulties, e.g., poor therapeutic efficacy and high recurrence rate. Using nanocarriers as drug delivery systems has been extensively researched both experimentally and clinically, and the outstanding properties of nanocarriers drug delivery systems, e.g., high drug capacity, adjustably specific targeting capability, stable structure, etc., endow nanocarriers with high performance with great application potential in treatment and diagnosis of cancer such as esophageal cancer, breast cancer, liver cancer, and so on. The relationship between the characteristics, synthesis, and properties of nanocarriers are relevant to their therapeutic/diagnosis performance. It has been proved that the characteristics and synthesis methods of different nanomaterials play a crucial role in determining the size, structure, and transport capabilities of these nanocarriers, which is critical factor to design and construct the optimal drug delivery systems (Lee et al., 2017). The nanoscale dimensions and controllably modified property of nanocarriers in these delivery systems present significant advantages in enhancing both the pharmacokinetics and pharmacodynamics of drugs and further improve treatment outcomes (Bozzuto and Molinari, 2015). Notably, extensive advancements have been made in the field of nanocarriers for drug delivery systems, showcasing their immense potential in prolonging drug circulation time, improving bioavailability, enhancing drug accumulation at tumor sites, reducing drug resistance, minimizing adverse reactions associated with anticancer medications, etc. (Figure 1) (Patra et al., 2018).

To facilitate effective treatment and diagnosis of cancer, the construction of nanocarriers for drug delivery systems should adhere to several crucial criteria (Tang et al., 2017), including:

1) Safety: Nanocarriers must be composed of non-toxic and biocompatible materials to ensure their safety and compatibility within biological systems.
2) Size: The size of nanocarriers should be appropriate to enhance passive targeting efficiency to the bone marrow. While some researchers have proposed that the optimum size of nanocarriers should be smaller than 150 nm (Moghimi, 1995), a universally agreed-upon standard has yet to be established.
3) Capacity: Nanocarriers should possess an adequate drug-loading capacity to achieve therapeutic doses.
4) Stability: To accomplish targeted delivery and therapeutic tasks, the nanocarriers should maintain a period of circulation time within the physiological system. Thus, the nanocarriers should be tolerant in the physiological environment, i.e., the stability, surface functionalization, and dispersity of nanocarriers must withstand in drug effective time resisting aggregation induced by solution acidity/alkalinity, temperature, ionic strength, and interactions with macromolecules. Moreover, the excellent stability of nanocarriers ensures the prevention of drugs rapid degradation or clearance within the bloodstream.
5) Elimination: Nanocarriers should possess appropriate clearance mechanisms after fulfilling their intended functions to avoid long-term accumulation and triggered systemic adverse effects. Meanwhile, timely eduction can effectively ease the excessive burden on excretory organs.
6) Targeting: Targeting capabilities of nanocarriers consist of active targeting and passive targeting progress. The highly specific targeting capabilities can allow the drug to preferentially accumulate at the diseased site while minimize adverse effects on healthy tissues.
7) Adjustability: Leveraging the characteristic differences between cancer cells and normal cells in terms of extracellular and intracellular environments (such as pH, redox potential, and enzymatic activity), responsive nanocarriers can be stimulated to facilitate the controlled release of encapsulated drugs specifically within the target tissue and cells. Therefore, the design of suitable nanocarriers can exploit the unique physiological conditions associated with the disease.

By meeting these stringent criteria, nanocarriers for drug delivery systems hold great promise for improving cancer therapy and diagnosis. Even though the correlational researches about nanocarriers applied in drug delivery systems are explored and developed ceaselessly, the systematic overview about the nanocarriers used as drug delivery systems for therapeutic and diagnosis of esophageal cancer is still lack. Herein, we reviewed some instructive researches about the application of nanocarriers drug delivery systems in therapeutic and diagnosis of esophageal cancer and its complications. In brief, the representative cases about nanocarriers drug delivery systems used in esophageal cancer, i.e., diagnosis, chemotherapy, targeted drug, radiotherapy, immunity, surgery, and new therapeutic method, were generalized while their applications in esophageal stenosis or obstruction and oesophagitis were also summarized, and the nanomaterials used in Chinese patent medicine were firstly overviewed (Figure 1). Moreover, we also discussed the challenge and the future of nanocarriers drug delivery systems in the end. Hence, this omnibearing review is significative to drive the development of therapeutic and diagnosis of esophageal cancer, and may be enlightening for other cancer therapeutic.

## 2 The applications of nanocarriers in therapeutic and diagnosis of esophageal cancer

**FIGURE 2:**
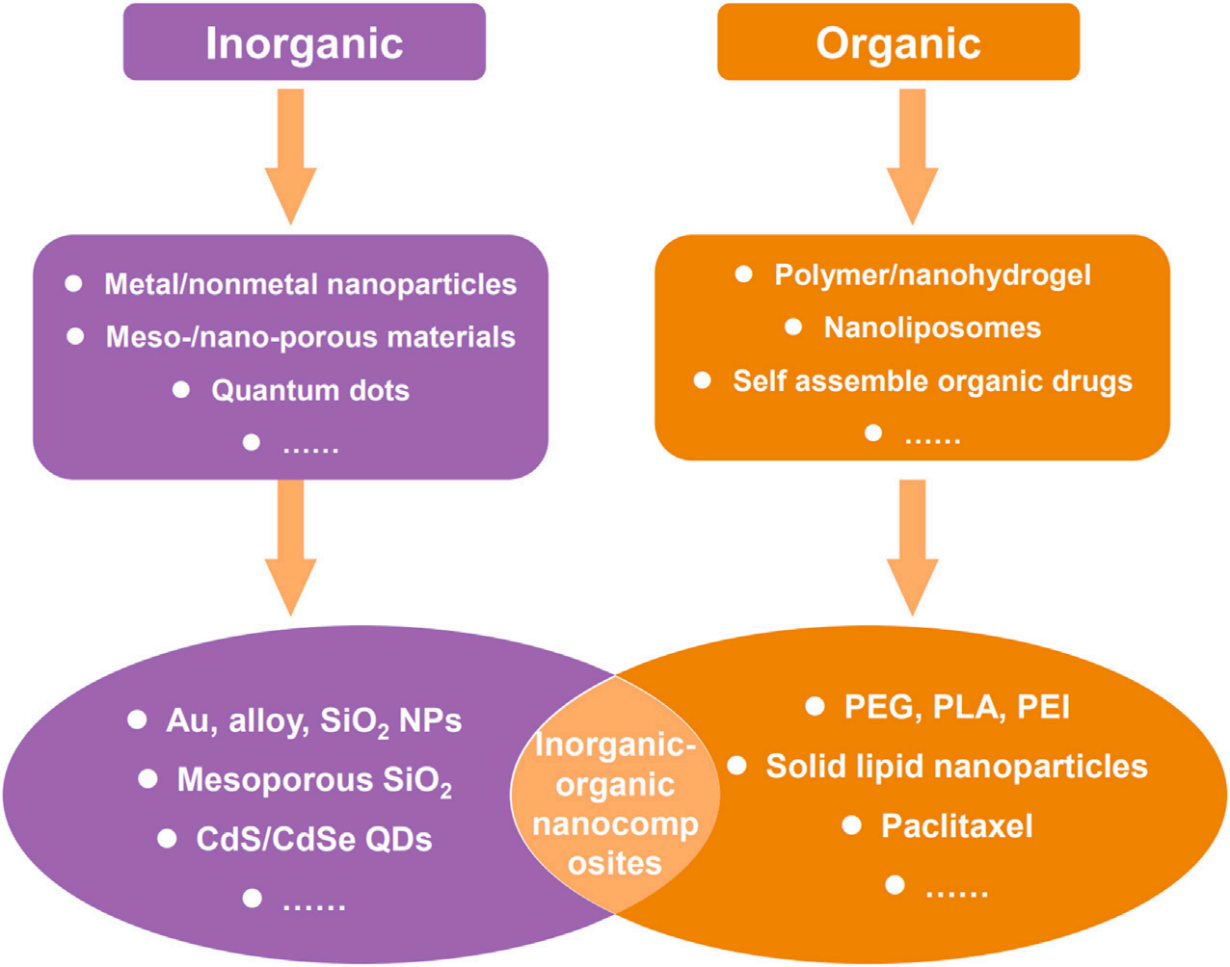
Common two types of nanomaterials for therapy and diagnosis of cancer, and some typical examples.

Nanocarriers employed in drug delivery systems can be broadly categorized into two major classes: organic nanocarriers and inorganic nanocarriers. Organic nanocarriers encompass polymers, liposomes, and proteins, and inorganic nanocarriers include various nanoparticles such as gold nanoparticles, silicon nanoparticles, iron nanoparticles, selenium nanoparticles, copper nanoparticles, and other inorganic salt nanoparticles. Both organic nanocarriers and inorganic nanocarriers possess different characteristics but equal importance in drug delivery systems, so there are also many examples using organic-inorganic nanocomposite as nanocarriers to realize functional complementarity (Figure 2). Thus, to roundly summarize and analyze the effect of nanocarriers in the therapeutic and diagnosis of esophageal cancer, all of organic nanocarriers, inorganic nanocarriers, and composite nanocarriers were reviewed in subsequent content.

### 2.1 The applications of nanocarriers in esophageal cancer diagnosis

When using nanocarriers as a diagnostic tools for esophageal cancer, they are typically prepared as nanoprobes for direct diagnosis or utilized for enhancing existing diagnostic measures through their unique properties. As for direct diagnosis, the fluorescent substances are combined with nanoparticles or nanocapsules to prepare imaging probes with high contrast and high resolution which can be used to detect tumors, cell activity, and vascular activity. Furthermore, for another way, i.e., using nanocarriers to enhance/assist diagnosis, the nanocarriers are always introduced into existing diagnostic measure such as computerized tomography (CT), nuclear magnetic resonance (NMR) imaging, Surface-Enhanced Raman Scattering (SERS), etc. For example, magnetic nanoparticles can be prepared as nanoprobes for magnetic resonance imaging, and these probes can improve image contrast and sensitivity, thus allowing for better detection of tumors and other diseases.

Li et al. established a novel method based on resonance Rayleigh scattering (RRS) to detect the overexpression of epidermal growth factor receptor (EGFR) in esophageal cancer cells. In this strategy, a multifunctional gold nanoparticle probe (Apt-Au nanoparticles-Ab) was developed by multi-functionalizing Au nanoparticles with aptamers and anti-EGFR antibodies which was subsequently delivered into EGFR positive cancer cell. The diagnostic results show that, remarkably, the RRS intensity significantly increased upon mixing the probe with Eca-109 esophageal cancer cells meaning that this RRS-based detection platform provides a valuable tool for identifying EGFR-positive cancer cells and exhibits substantial potential for clinical diagnostics (Li et al., 2021). Gai et al. conducted research on the development of a novel targeted dual-specific antibody system encapsulated in chitosan-Fe_3_O_4_ nanoparticles, specifically targeting fibroblast growth factor receptor (FGFR) and vascular endothelial growth factor receptor (VEGFR). As a result, both *in vivo* and *in vitro* experiments, this system demonstrated its ability to enhance the resolution of CT imaging and improve the accuracy of diagnosis for early detection and final confirmation of suspected cases (Gai et al., 2018). Moreover, Duo et al. employed surface-enhanced Raman scattering (SERS) based on silver nanoparticles to analyze and classify SERS spectra obtained from both normal and cancerous plasma samples, and the diagnostic accuracy achieved was notably high, approximately 90%, making this approach holds promising potential as a label-free and straightforward blood test for the detection of esophageal cancer (Lin et al., 2014). The magnetic nanocarriers are frequently used to improve diagnostic accuracy through enhancing image contrast and sensitivity in NMR imaging process. The introduction of gadolinium-iron nanoparticles as drug delivery carriers is associated with various parameters (e.g., nanoparticle size, hydrophobicity/hydrophilicity, surface charge, core composition, coating properties, administration route, and dosage) that influence their biodistribution. Because of this particular phenomenon and the magnetic properties of these nanoparticles, the real-time monitoring of drug delivery and treatment responses can be realized (Alphandéry, 2019). Li et al. developed a polyethylene glycol-b-poly (1-(3-aminopropyl)-3-(2-methacryloyloxypropyl)imidazolium bromide) (PAMPImB) complex carrier for DNA loading and magnetofection which consists of four layers, i.e., Fe_3_O_4_ nanoparticle core, Au shell, internal PAMPImB block, and PEG corona. In this four layers structure, each layer is designed to perform different functions: the Fe_3_O_4_ nanoparticle core can facilitate cellular uptake through magnetic acceleration; the Au shell promotes copolymer binding via Au-S bonds; the internal PAMPImB blocks for DNA condensation; and the PEG corona enhances colloidal stability. Transfection efficiency studies conducted on human esophageal cancer cells (EC-109) revealed that the nanocomplex exhibited high transfection efficiency within a shorter incubation time when subjected to an external magnetic field, which was caused by enhanced cellular uptake through magnetic acceleration, providing a feasible method for rapid cancer diagnosis (Li et al., 2017). Furthermore, Fe_3_O_4_ nanoparticle-modified CdSe/CdS/ZnS nanocrystals can be utilized for the immediate optomagnetic detection of cancer biomarkers, including esophageal cancer, in serum samples (Qureshi et al., 2020). In addition, the clinical trials for cancer diagnosis and treatment using nanodrugs based on magnetic iron oxide nanoparticle were shown in Table 1.

**TABLE 1:** The clinical trials for cancer diagnosis and treatment using nanodrugs based on magnetic iron oxide nanoparticle (by May 2023; Source: clinicaltrials.gov) (Liang et al., 2020).

Project ID	Methods	Cancer type	Stage	Start/end date	State
NCT02689401	MRI to detect lymph node metastases	Esophageal cancer	Phase 1	2016/2016	Withdrawn
NCT02857218	MRI before neoadjuvant chemoradiation therapy and again before esophagectomy	Esophageal cancer (Stage IIB-IIIC)	Early Phase 1	2018/2021	Withdrawn
NCT02253602	Innovative MRI Techniques to Improve Treatment Stratification of Patients With Esophageal Cancer	Esophageal Neoplasms	Phase 1	2014/2018	Completed

### 2.2 The applications of nanocarriers in chemotherapy of esophageal cancer

Chemotherapy is a crucial systemic treatment for esophageal cancer, and currently, commonly used chemotherapy drugs include paclitaxel, platinum agents, and fluoropyrimidine drugs. However, these chemotherapy drugs exhibit similar concentrations in both plasma and tumor tissues, leading to various toxic side effects that render long-term chemotherapy intolerable for patients. Moreover, tumor blood vessels exhibit distinct pathological and physiological characteristics that are not typically observed in normal vasculature, including substantial increase in proliferating endothelial cells, enlarged and tortuous structures, the absence of pericyte coverage, and abnormal basement membranes. Thus, the therapeutic efficacy of traditional chemotherapy drugs may be weakened while the therapeutic process may be accompanied by the adverse side effect causing harmful consequence. Given these unique traits, nanoparticle-based drug delivery systems hold great potential for the chemotherapy of esophageal cancer. Nanocarriers drug delivery systems possess several distinguishing properties that set them apart from conventional cancer therapies, such as 1) the ability to act as both therapeutic agents and diagnostic tools for cancer, which can realize real-time monitoring of drug delivery and treatment responses; 2) the capacity to encapsulate multiple anti-cancer drug molecules, enabling synergistic therapeutic effects; 3) the potential to enhance specificity to tumor cells through passive and active targeting mechanisms; 4) the ability to achieve controlled drug release, thereby increasing drug concentrations within cancer cells while reducing toxicity to normal cells, ultimately leading to improved therapeutic outcomes and reduced systemic toxicity. Hence, using nanocarriers as a chemotherapeutic drug delivery systems can effectively maximize drug efficacy, prolong drug action time, and reduce harmful side effects.

#### 2.2.1 The targeting process of nanocarriers in chemotherapy

The targeting process of nanocarriers can be divided into two types, i.e., passive targeting and active targeting, and these two types of targeting process experience different mechanisms. For passive Targeting, the enhanced permeability and retention effect (EPR) arises from the formation of leaky blood vessels and impaired lymphatic drainage in rapidly growing tumors, which contributes to the accumulation of nanoparticles and microparticles within the tumor. Extensive research has been conducted on nanosized drug carriers, such as liposomes, dendrimers, polymer-drug conjugates, polymer micelles, and inorganic nanoparticles, for drug delivery in cancer chemotherapy using this distinctive approach (Akhter et al., 2018). Due to their specific size range (typically between 1 nm and 200 nm), these nanoparticles preferentially accumulate at the tumor site by exploiting the highly permeable blood vessels and leveraging the EPR effect (Liao et al., 2020)]. Distinguishing with passive targeting, the active targeting employs surface-conjugated ligands that interact with cell membrane receptors, and nanocarriers facilitate receptor-mediated endocytosis, leading to increased drug concentrations within the target cells (Hejmady et al., 2020). Tumor cells often exhibit overexpression of specific receptors, making these receptors ideal targets for active targeting using ligand-functionalized nanoparticles (Juan et al., 2020). Consequently, both tumor cells and endothelial cells are considered viable cellular targets for active targeting strategies. Additionally, nanoparticles have the potential to address these limitations by leveraging the EPR effect to accumulate anticancer drugs specifically within tumor tissues, and nanoparticles can prolong the half-life of drugs, enhance the solubility of hydrophobic drugs, and reduce potential immunogenicity (Shi et al., 2010). Thus, through active targeting, nanocarriers can mitigate the non-specific distribution of drugs, enhance drug accumulation within the tumor, and improve treatment safety and efficacy.

#### 2.2.2 The applications of nanocarriers combined with chemotherapy drugs used in chemotherapy

Dohmitsu et al. conducted a study to evaluate the antitumor activity of TAC-1043, a thermosensitive liposome containing encapsulated cisplatin within large unilamellar vesicles composed of a 9:1 ratio of dipalmitoyl phosphatidylcholine and distearoyl phosphatidylcholine, and the results demonstrated that the combined administration of TAC-1043 and hyperthermia significantly inhibited tumor cell proliferation (Dohmitsu et al., 1991). Wang et al. developed a novel nanoplatform for the treatment of esophageal cancer based on ATP-responsive drug release. The chemotherapeutic drug doxorubicin was inserted into an ATP aptamer (Ap) to form a double-stranded DNA (“DNA duplex”) which was subsequently condensed using polyethyleneimine (PEI) to construct the final nanoplatform (PEI-Ap-DNA-DOX). After internalization by cancer cells, the doxorubicin-loaded DNA duplex can be opened and released within the intracellular environment rich in ATP (Wang et al., 2020c). Similarly, Huang et al. developed a novel formulation of docetaxel, called trimethyl chitosan-DTX (TMC-DTX) applying to the treatment of esophageal cancer, and investigated its effects. TMC-DTX exhibited remarkable inhibition of cell proliferation and promoted apoptosis in EC cells, effectively reducing tumor volume in a xenograft mouse model (Huang et al., 2015). Moreover, solid lipid nanoparticles (SLNs) coated with trimethyl chitosan were prepared using ultrasonication and thermal shear homogenization techniques for the encapsulation of erlotinib with a three layers structure, i.e., trimethyl chitosan-coated erlotinib-loaded SLNs (TMC-IRN-SLN). The presence of trimethyl chitosan on the SLN surface facilitated electrostatic interactions with the lipid layer and increased the particle internalization, resulting in sustained release of the drug in an acidic environment, and these nanoparticles exhibited excellent cytotoxicity against EC9706 cells, leading to an increased proportion of apoptotic cells in both early and late-stage apoptosis. This formulation aimed to enhance stability, improve drug payload, and achieve prolonged drug release in esophageal cancer cells (Ji et al., 2018).

NK012 is a novel micellar nanoparticle formulation containing SN-38, an analogue of irinotecan, and it exhibits superior anti-esophageal cancer activity and induces longer survival compared to traditional CPT-11, with a lower clearance rate. The experimental result shows that the concentration of polymer-bound SN-38 and released SN-38 in tumor tissue remains sustained for an extended period. Thus, with its specific distribution and prolonged release of SN-38 within the tumor, NK012 may represent a promising approach for treating esophageal cancer by exerting time-dependent antitumor activity (Hamaguchi et al., 2010). In a phase I clinical trial conducted by Hamaguchi et al., the anticancer efficacy of NK012 was evaluated in adult patients with solid tumors, and three patients with esophageal cancer participated in the trial, and one refractory patient achieved an objective response and continued the study treatment for 5 months (Hamaguchi et al., 2010). The recommended dose of NK012 was determined to be 28 mg/m^2^, administered every 3 weeks as a treatment cycle. Recently, an open-label phase I clinical trial evaluated the safety and efficacy of eribulin liposomes in Japanese patients with advanced solid tumors, including esophageal cancer. The findings indicated that eribulin liposomes administered at a dose of 2.0 mg/m^2^ every 3 weeks were well tolerated, suggesting the need for further large-scale investigations in these patient populations (Udagawa et al., 2023).

Recently, Liu et al. prepared a chemotherapy drug based on inorganic Au nanomaterials and Rhus coriaria L. fruit aqueous extract. This composite drug can treat several types of esophageal cancer, such as human esophageal squamous cell carcinoma, human Caucasian esophageal carcinoma, adenocarcinoma of the gastroesophageal junction, and distal esophageal adenocarcinoma. The excellent antioxidant and anti-esophageal cancer activities let this composite drug indicate suitable antitumor property without any cytotoxicity effect on the normal cell (Liu J. et al., 2020). Moreover, more and more natural extracts have been used in chemotherapy.

#### 2.2.3 The applications of nanocarriers based on paclitaxel used in chemotherapy

**FIGURE 3:**
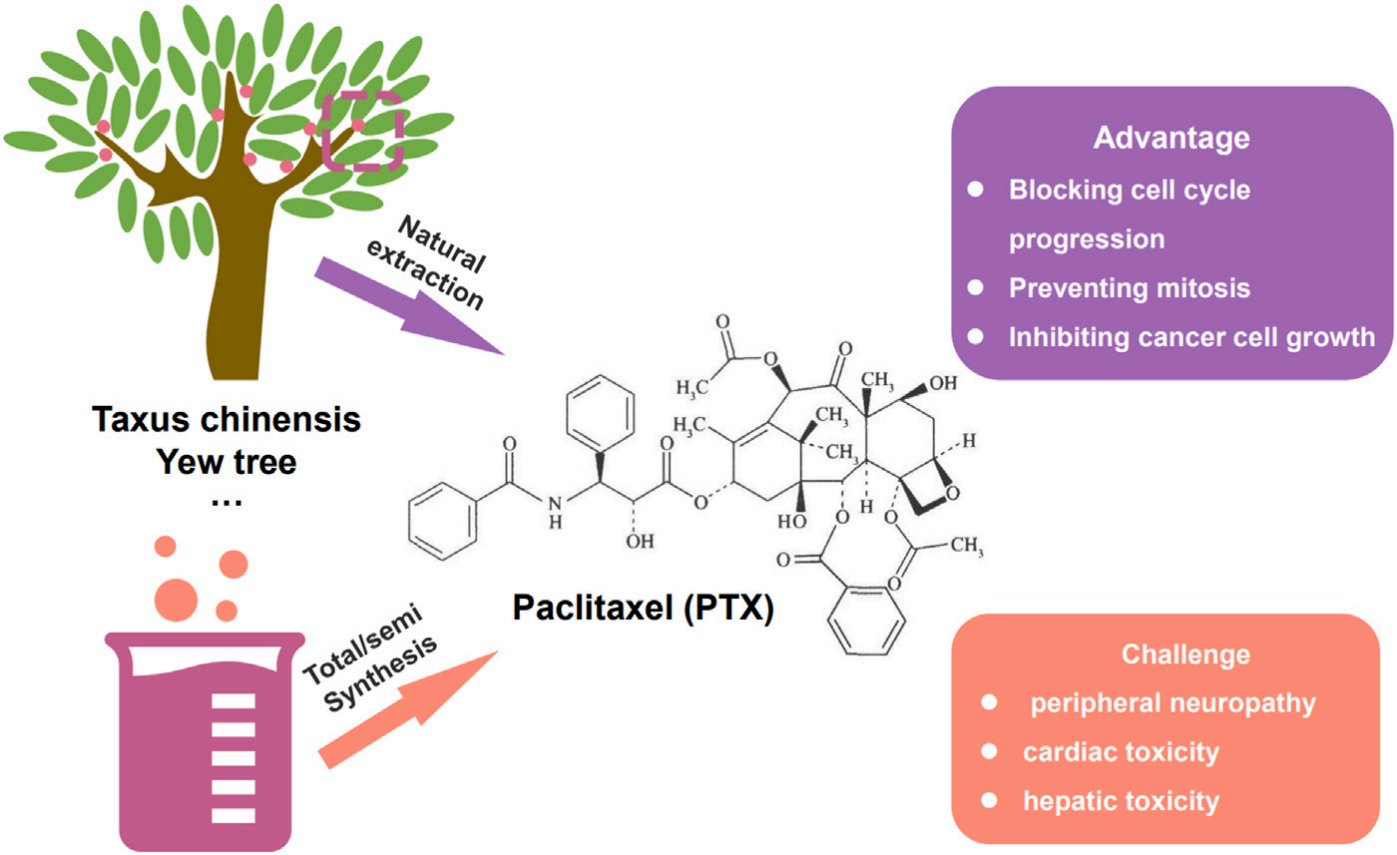
The way to gain paclitaxel (PTX), i.e., natural extraction and total/semi synthesis. The advantage and existing challenge of paclitaxel.

Paclitaxel (PTX) is a naturally derived compound widely used in the treatment of various types of cancer due to its unique mechanism of action, which involves blocking cell cycle progression, preventing mitosis, and inhibiting cancer cell growth. However, the administration of PTX is associated with potential adverse effects such as peripheral neuropathy, cardiac toxicity, and hepatic toxicity, which are major drawbacks of this highly effective drug (Figure 3). Specifically, peripheral neuropathy can manifest as shooting or burning pain (particularly in the hands and feet), sensory loss, numbness, and tingling sensations (Wang et al., 2017). To address the adverse issues of single PTX, several nanocarrier formulations have been developed to mitigate the adverse effects of PTX and improve its water solubility without the commonly used solvent Cremophor^®^ EL which has been associated with toxicity (Kiss et al., 2013) (Table 2). Genexol-PM^®^ is a polyethylene glycol (PEG)-b-poly (D,L-lactide) (PEG-PLA) micellar formulation of PTX, approved in 2007 in Korea for the treatment of breast cancer and non-small cell lung cancer. This formulation exhibits lower toxicity compared to PTX formulated with Cremophor^®^ EL, with a maximum tolerated dose determined to be 2 to 3 times higher (Table 2). Besides, Nanoxel^®^, a polymer-based amphiphilic micellar formulation of PTX, has received clinical approval in India in 2006 and is currently undergoing FDA-approved clinical trials (ClinicalTrials.gov Identifier: NCT04066335), and Apealea, approved for use in the European Union in 2018, utilizes a PTX conjugate with poly-l-glutamic acid. However, despite these formulation advancements, peripheral neuropathy remains a clinical challenge, emphasizing the need for further optimization (Riedel et al., 2021).

**TABLE 2:** Representative commercial materials for PTX applications.

Materials	Trade name	Characteristic	Application
polyoxyethylated castor oil	Cremophor^®^ EL	toxicity	
PEG-*b*-PLA/PTX micellar	Genexol-PM^®^	lower toxicity (with a maximum tolerated dose determined to be 2 to 3 times higher)	breast cancer and non-small cell lung cancer
mPEG-b-PDLLA/PTX amphiphilic micellar	Nanoxel^®^	lower toxicity and hydrosoluble	breast cancer, ovarian cancer, and non-small cell lung cancer

Researchers have discovered that the expression level of PI3K is higher in ESCC patients compared to normal individuals, so by using nanoparticles loaded with a PI3K inhibitor (AZD8186) and docetaxel (DTX) on esophageal squamous cell carcinoma cells, synergistic effects between nanoparticles and drugs have been observed, along with a significant reduction in hematological toxicity (Wang et al., 2018). Some scholars conducted *in vitro* studies, as well as subcutaneous tumor transplantation and peritoneal metastasis tumor survival models in mice with esophageal adenocarcinoma, and found that compared to conventional paclitaxel, nanoparticle-bound albumin-paclitaxel showed significantly improved *in vivo* antitumor efficacy and overall survival rates, whether used alone or in combination therapy (Hassan et al., 2017). In another study, Cui et al. developed albumin-based microspheres incorporating magnetic Fe_3_O_4_ nanoparticles as carriers for paclitaxel. These microspheres demonstrated excellent targeting ability and sustained release of paclitaxel, effectively inhibiting the growth of Eca109 cells (Cui et al., 2012).

Nowadays, the combination regimen of nab-paclitaxel and cisplatin has emerged as an effective and well-tolerated first-line treatment option treatment option for esophageal cancer in China (Wu et al., 2017). Shi et al. conducted a clinical study to investigate the efficacy and safety of nab-paclitaxel combined with cisplatin in patients with metastatic esophageal cancer, which contained a total of 33 patients with metastatic disease were included in the study (Shi et al., 2013). The results demonstrated significant efficacy of the nab-paclitaxel plus cisplatin regimen, with a disease control rate of 87.9%, an objective response rate of 60.0%, a median overall survival of 15.5 months, and a median progression-free survival of 6.2 months. Nab-paclitaxel, a novel nanoparticle-based drug, is a 130 nm albumin-bound formulation of paclitaxel that offers enhanced water solubility and solvent-free delivery of paclitaxel (Yuan et al., 2015), and unlike conventional paclitaxel formulations, nab-paclitaxel utilizes albumin as a stable carrier for paclitaxel, eliminating the need for toxic solvents such as ethanol and polyoxyethylated castor oil (Cremophor^®^ EL) (Zhao et al., 2022). These solvents have been associated with severe side effects, including peripheral neuropathy and hypersensitivity reactions while the use of solvents can limit the drug’s efficacy and bioavailability. Therefore, the solvent-free nab-paclitaxel represents a promising advancement in paclitaxel therapy, offering reduced adverse reactions and improved therapeutic outcomes. A study by Hassan et al. investigated the anti-tumor effects of nab-paclitaxel on esophageal cancer cells through *in vitro* and *in vivo* experiments revealing that nab-paclitaxel significantly suppressed the proliferation of esophageal cancer cells and induced higher rates of apoptosis compared to paclitaxel alone or the control group (Hassan et al., 2018).

Additionally, in mouse models of subcutaneous xenograft and peritoneal metastasis, nab-paclitaxel exhibited a significant increase in survival rates. Overall, nab-paclitaxel and albumin-based paclitaxel formulations offer promising prospects for the treatment of esophageal cancer, with improved therapeutic efficacy and reduced adverse effects. Further exploration and optimization of these formulations are necessary to fully realize their potential in clinical applications. While nab-paclitaxel has been widely utilized as a nanomedicine in the treatment of esophageal cancer, research exploring the application of other nanocarriers, such as viral capsids and dendrimers, in this context remains limited and requires further investigation (Li et al., 2022). The representative nanomaterials used in chemotherapy of esophageal cancer were summarized in Table 3.

**TABLE 3:** Representative nanomaterials used in chemotherapy of esophageal cancer.

Materials	Types	Features and findings	Application	References
PEI-Ap-DNA-DOX	Organic	ATP-responsive drug release	esophageal cancer	Wang et al. (2020c)
Trimethyl chitosan-DTX	Organic	Remarkable inhibition of cell proliferation and promoted apoptosis in EC cells, effectively reducing tumor volume in a xenograft mouse model	esophageal cancer	Huang et al. (2015)
Trimethyl chitosan-coated erlotinib-loaded solid lipid nanoparticles	Organic	Excellent cytotoxicity against EC9706 cells	early and late-stage apoptosis	Ji et al. (2018)
Nanoparticles loaded with a PI3K inhibitor (AZD8186) and docetaxel (DTX)	Organic	Significant reduction in hematological toxicity	esophageal squamous cell carcinoma cells	Wang et al. (2018)
Nanoparticle-bound albumin-paclitaxel	Organic	significantly improved *in vivo* antitumor efficacy and overall survival rates	esophageal adenocarcinoma	Hassan et al. (2017)
Albumin-based microspheres incorporating magnetic Fe_3_O_4_ NPs as carriers for paclitaxel	Inorganic-organic nanocomposites	excellent targeting ability and sustained release of paclitaxel, effectively inhibiting the growth of Eca109 cells	esophageal cancer	Cui et al. (2012)
Nab-paclitaxel combined with cisplatin	Inorganic-organic nanocomposites	significant efficacy of the nab-paclitaxel plus cisplatin regimen, with a disease control rate of 87.9%, an objective response rate of 60.0%, a median overall survival of 15.5 months, and a median progression-free survival of 6.2 months	esophageal cancer	Shi et al. (2013)

### 2.3 The applications of nanocarriers in radiotherapy of esophageal cancer

Concurrent chemoradiotherapy is an essential modality for the local treatment of unresectable esophageal cancer, but due to the anatomical position of esophagus is in close proximity to vital organs such as the lungs, heart, and spinal cord, it presents challenges in escalating the local radiation dose without causing adverse effects like radiation-induced lung injury and cardiac toxicity. Furthermore, although chemotherapy drugs are used as radiosensitizers to synergize with radiation therapy, suboptimal outcomes are normally observed due to similar drug concentrations in both plasma and tumor tissue. Additionally, the majority of esophageal cancer patients are at risk of developing malnutrition, and during radiation therapy, they are susceptible to life-threatening complications, including perforation, bleeding, and tracheoesophageal fistula formation, which magnifies the adverse side effects of chemotherapy drugs and radio. Consequently, achieving satisfactory precisely local control of radiotherapy with minimized adverse side effect remains a challenge, and considering these limitations, the exploration of novel breakthroughs in the field of radiation therapy for esophageal cancer is in an urgent demand to improve therapeutic efficacy and simultaneously alleviate patient’s pain and sufferings.

#### 2.3.1 The applications of organic nanocarriers used in radiotherapy

The above-mentioned paclitaxel is also commonly used as a radiosensitizer in the treatment of various tumors, and the application of paclitaxel in (chemo) radiotherapy shows great potential in cancer therapy. Moreover, more and more researchers put their attention on improving the radiotherapy effectiveness of paclitaxel to achieve better therapeutic efficacy. A novel approach has been developed by linking recombinant protein anti-EGFR and internalizing arginine-glycine-aspartic acid (IRGD) to the surface of paclitaxel-loaded red blood cell membrane nanoparticles (IE-PR nanoparticles). This innovative nanoscale radiosensitizer has demonstrated increased sensitization rates of 1.32-fold in EGFR-overexpressing esophageal cancer cells and 1.25-fold in EGFR-low-expressing cells compared to paclitaxel alone suggesting that this strategy has the potential to overcome the current limitations in esophageal cancer treatment (Ai et al., 2018). In a similar vein, some researchers have also explored the use of modified with anti-EGFR-iRGD fusion protein red blood cell membrane nanoparticles loaded with paclitaxel. *In vitro* tumor studies have shown that these nanoparticles enhance the radiosensitizing effects by effectively arresting more tumor cells in the radiation-sensitive G/M phase, which leads to an increased production of reactive oxygen species and the formation of more lethal DNA double-strand breaks (Ren et al., 2018). In a retrospective clinical study conducted at a single center, the efficacy and feasibility of liposomal paclitaxel combined with cisplatin in the treatment of locally advanced squamous cell carcinoma of the esophagus were evaluated. The results suggested that the use of liposomal paclitaxel in combination with cisplatin for chemoradiotherapy yielded good tolerability and effectiveness in managing locally advanced squamous cell carcinoma of the esophagus (Yi et al., 2022). Besides, another retrospective clinical study analyzed the efficacy and safety of neoadjuvant chemotherapy based on liposomal paclitaxel in conjunction with platinum in patients with locally advanced resectable esophageal squamous cell carcinoma. The study demonstrated that a satisfactory R0 resection rates, survival rates, and significant tumor downstaging effects can be achieved while maintaining a good safety profile (Wang et al., 2022). In a single-center retrospective clinical study, the use of liposomal paclitaxel and cisplatin in synchronous chemotherapy was found to improve the prognosis of patients with locally advanced esophageal squamous cell carcinoma undergoing intensity-modulated radiotherapy (Liu et al., 2019). The therapeutic value of combined chemoradiotherapy in the treatment of locally advanced esophageal squamous cell carcinoma still require larger prospective studies to validate.

Due to the limited retention time of radiation in tumor tissues the radiotherapy effectiveness of the traditional *in vivo* irradiation techniques has been hampered, but by leveraging nanodrug delivery technology, it is possible to enhance the targeted action of radioisotopes, resulting in improved local control rates and reduced adverse reactions. In a preclinical animal model of esophageal cancer, Chang et al. have employed rhenium-188 (188 Re) nano-liposomes in combination with external beam irradiation (beta radiation), which show an effective inhibition of the growth of tumor tissues without significant adverse reactions or biological toxicity (Chang et al., 2015). The combination of external beam radiation therapy and 188Re-liposomes exhibited a greater inhibitory effect on tumor regeneration compared to each treatment alone. Furthermore, this combined therapy showed no significant adverse effects or notable biotoxicity on parameters such as white blood cell count, body weight, or liver and kidney function. Even though iodine-125 (^125^I) brachytherapy has emerged as a highly effective palliative treatment option for advanced esophageal cancer, the development of resistance to ^125^I brachytherapy due to tumor hypoxia and activation of the hypoxia-inducible factor 1 (HIF-1) signaling pathway poses a significant limitation in the management of esophageal cancer. To overcome these challenges, Yao and colleagues have devised a novel approach to enhance the radiosensitivity of brachytherapy by co-encapsulating catalase (CAT) and the HIF-1 inhibitor, i.e., acriflavine (ACF), within the hydrophilic cavity of liposomes, creating a formulation termed (ACF-CAT@Lipo). ACF-CAT@Lipo exhibits remarkable properties when stimulated by overexpressed H_2_O_2_ in the tumor microenvironment and generates a significant amount of O_2_ both *in vitro* and *in vivo*, effectively alleviating tumor hypoxia. Furthermore, the synergistic effect of ACF substantially reduces the expression of hypoxia-related proteins, including HIF-1α, VEGF, and MMP-2. More importantly, this combined effect enhances the radiosensitivity of ^125^I brachytherapy, leading to improved therapeutic outcomes and potential eradication of esophageal cancer *in vivo* (Yao et al., 2022).

#### 2.3.2 The applications of inorganic nanocarriers used in radiotherapy

Moreover, a novel nanomaterial composed of graphdiyne (GDY) anchored with cerium oxide (CeO_2_) nanoparticles was also used, known as GDY-CeO_2_ nanocomposites. This unique composite material demonstrates exceptional catalase activity in decomposing H_2_O_2_ into O_2_, offering a significant advantage in mitigating tumor hypoxia. In addition, the GDY-CeO_2_ nanocomposites promote radiation-induced DNA damage, thereby inhibiting tumor growth and potentially reversing radioresistance in esophageal cancer treatment (Zhou et al., 2021). A pH-responsive nano-system possessing capability of aggregating and carrying doxorubicin (DOX) has been developed for enhanced radiosensitization and synergistic radiochemotherapy in esophageal cancer. Within the acidic microenvironment of the tumor, the size of nano-aggregates increase and the reflux of gold nanoparticles (Au NPs) into the bloodstream can be impeded. Consequently, tumor accumulation and retention of Au NPs are enhanced, resulting in higher concentrations and larger sizes of Au NPs within the tumor, and in turn, significantly improves the radiosensitizing effect. Simultaneously, the DOX payload is selectively delivered and released into tumor cells triggered by the acidic microenvironment as a synergistic approach to radiochemotherapy. *In vitro* experiments have demonstrated that the pH-responsive Au NPs intensify radiation-induced DNA damage, promote cell apoptosis, induce cell cycle arrest, and reduce colony-forming ability. Notably, *in vivo* studies have also shown a marked enhancement in the anti-tumor efficacy (Luan et al., 2022). The representative nanomaterials for radiotherapy of esophageal cancer were summarized in Table 4.

**TABLE 4:** Representative nanomaterials for radiotherapy of esophageal cancer.

Crucial component	Materials	Features and findings	Application	References
miRNA	Sulfuric fish gelatin nanoparticles as carriers for miR-203	Targeting specific molecules such as Ran and DNp63, increasing local drug concentrations, and efficiently suppressing tumor proliferation and invasion	esophageal squamous cell carcinoma	Cao et al. (2013)
siRNA	PEI coated mesoporous Si NPs loaded with siRNA by EC-9706 tumor cells	Significant inhibition of EC-9706 tumor cells proliferation	esophageal cancer	Sun et al. (2017)
	Carboxymethyl chitosan-auristatin and siRNA	Targeting MVP and BCL2, inhibited cancer cell growth and tumor development in both cultured esophageal squamous cell carcinoma cells and xenograft models of mice	esophageal squamous cell carcinoma	Zhang et al. (2022)
polypeptide	NGR/PEI/TANOL	Pronounced enhancement in the uptake by well-differentiated human esophageal adenocarcinoma cells	esophageal cancer	(MOFFATT and WANGARI)
small molecule	Reversible disulfide crosslinked micelles-docetaxel and the PI3K inhibitor AZD8186	Minimizing premature release into the bloodstream and reducing nonspecific toxicity, synergistic antitumor effect of the DCM-loaded docetaxel and AZD8186	esophageal squamous cell carcinoma	Wang et al. (2017)

### 2.4 The applications of nanocarriers in targeted therapy of esophageal cancer

In recent years, molecular targeted therapy has emerged as a significant breakthrough in the treatment of various malignant tumors, particularly lung cancer, colorectal cancer, and liver cancer. In terms of targeted therapy, for HER-2 positive advanced esophagogastric junction cancer, trastuzumab can be considered as a treatment option after the third-line of treatment. Targeted drugs against angiogenesis can also be used as treatment options, with apatinib being recommended for advanced esophagogastric junction cancer after the third-line of treatment. For second-line treatment of advanced esophageal squamous cell carcinoma, anlotinib or apatinib are viable options. However, the effective small molecule targeted drugs for esophageal cancer are scarce due to the high heterogeneity of esophageal tumor cells, making it challenging to effectively target them. miRNAs as a class of endogenous non-coding small RNA molecules play a crucial role in the development, progression, and metastasis of esophageal cancer, and they have become promising biomarkers for diagnosing and treating this disease (Chen et al., 2018; Pennathur et al., 2019). To address these challenges, researchers have explored the use of nanotechnology, specifically nanoparticle-based delivery systems, to enhance the effectiveness of small molecule targeted drugs in esophageal cancer treatment.

Research has identified abnormal expression levels of approximately 50 miRNAs in esophageal cancer tissues, among which about 7 miRNAs can distinguish between normal and malignant tissues. Notably, the downregulation of miR-203 in esophageal cancer suggests its close association with the initiation and progression of the disease (Zhang et al., 2019). The nanoparticles have been proved as a suitable carriers to load miRNA and achieve targeting delivery. For instance, utilizing sulfuric fish gelatin nanoparticles as carriers for miR-203, targeted therapy can be achieved to target specific molecules such as Ran and DNp63 in esophageal squamous cell carcinoma (ESCC), leading to the repair of tumor cells (Cao et al., 2013). This approach holds promise in effectively delivering therapeutic agents to esophageal tumor cells, increasing local drug concentrations, and efficiently suppressing tumor proliferation and invasion. Mesoporous silica nanoparticles (MSNP) synthesized through sol-gel method have been utilized as carriers which demonstrate the effective uptake of nano-complexes loaded with small interfering RNA (siRNA) by EC-9706 tumor cells, leading to a significant inhibition of cell proliferation *in vitro* experiments (Sun et al., 2017). Meanwhile, the positively charged polyethyleneimine (PEI) coating on the surface of silica nanoparticles can facilitate the binding with negatively charged ESCCAL-1 siRNA and effectively inhibit the growth of EC-9706 esophageal cancer cells. In addition, a novel nanoparticle carrier (NGR/PEI/TANOL) was developed by incorporating PEI into a TANOL nanoparticle formulation and subsequently modifying it with a tumor-targeting peptide, NGR. This composite exhibited a pronounced enhancement in the uptake by well-differentiated human esophageal adenocarcinoma cells, thereby facilitating more efficient targeting of tumor tissue *in vivo* (MOFFATT and WANGARI, 2018). These findings highlight the potential of utilizing nanoparticle carriers, whether loaded with small molecule targeted drugs or chemotherapy drugs, to effectively target heterogeneous esophageal tumor cells and provide a new paradigm for systemic treatment of esophageal cancer. In addition, Zhang et al. designed a multifunctional self-assembling nanoparticle platform based on carboxymethyl chitosan facilitating the simultaneous delivery of auristatin and siRNA targeting two genes associated with multidrug resistance in esophageal squamous cell carcinoma, i.e., MVP and BCL2. The synthesized nanoparticles successfully inhibited cancer cell growth and tumor development in both cultured esophageal squamous cell carcinoma cells and xenograft models of mice (Zhang et al., 2022).

Wang et al. used reversible disulfide crosslinked micelles (DCM) as targeted nanocarriers to deliver a potent combination of docetaxel and the PI3K inhibitor AZD8186, and for the first time, the effective inhibition of esophageal cancer cell growth in a mouse xenograft model. The use of DCM allowed for controlled drug release at the tumor site, minimizing premature release into the bloodstream and reducing nonspecific toxicity (Wang et al., 2017). The results showed a synergistic antitumor effect of the DCM-loaded docetaxel and AZD8186, highlighting their potential for targeted therapy of esophageal squamous cell carcinoma. More importantly, the study provided valuable insights into reducing systemic toxicity associated with the treatment (Li et al., 2022). In another work, folate-targeted paclitaxel-loaded micelles were evaluated for their efficacy in esophageal cancer treatment. When compared to free paclitaxel and conventional paclitaxel-loaded micelles, the folate-mediated polymer micelles exhibited superior effectiveness in inhibiting subcutaneous xenograft tumors and prolonging the survival of tumor-bearing nude mice. These findings suggest that folate-targeted micelles loaded with paclitaxel hold great promise as a potential therapeutic approach for human esophageal cancer (Wu et al., 2012). Additionally, Dai et al. developed a novel poly (ε-caprolactone)-Pluronic micelle system capable of encapsulating both doxorubicin and miR-34a, and the results demonstrated that the PCL-Pluronic micelles significantly enhanced the uptake of doxorubicin by esophageal cancer cells *in vitro*, leading to increased drug accumulation within the cells. The synergistic effect of the combined treatment of doxorubicin and miR-34a-loaded micelles was found to be superior to monotherapy, providing a promising strategy for improving the outcomes of esophageal cancer treatment (Dai et al., 2018)**.** The representative nanomaterials for target drugs of esophageal cancer were summarized in Table 5. Recently, a novel T7 peptide-modified pH-responsive targeting nano-system was developed by Deng et al., and in this nano-system the docetaxel and curcumin were co-loaded. As a result, T7-NP-DC was synthesized successfully with 100 nm diameter, good colloidal stability, and pH-responsive drug release property. The loading rate of docetaxel and curcumin is 10% and 6.1%, respectively, and good biosafety was observed, even when the concentration was high. In addition, the therapeutic efficacy of T7-NP-DC was better than NP-DC and docetaxel in terms of growth suppression in the KYSE150 esophageal cancer model (Deng et al., 2020).

**TABLE 5:** Representative nanomaterials used in target drugs of esophageal cancer.

Types	Materials	Features and findings	Application	References
Radiosensitizer	Linking recombinant protein anti-EGFR IRGD to the surface of paclitaxel-loaded red blood cell membrane NPs (IE-PR NPs)	Increased sensitization rates of 1.32-fold in EGFR-overexpressing esophageal cancer cells and 1.25-fold in EGFR-low-expressing cells compared to paclitaxel	esophageal cancer	Ai et al. (2018)
	Anti-EGFR-iRGD fusion protein red blood cell membrane nanoparticles loaded with paclitaxel	Enhance the radiosensitizing effects by effectively arresting more tumor cells in the radiation-sensitive G/M phase, lead to an increased production of reactive oxygen species and the formation of more lethal DNA double-strand breaks	esophageal cancer	Ren et al. (2018)
Chemoradiotherapy drugs	Liposomal paclitaxel combined with cisplatin	Good tolerability and effectiveness in managing locally advanced squamous cell carcinoma of the esophagus	esophageal squamous cell carcinoma	Yi et al. (2022)
Radioisotopes	188 Re nano-liposomes	effective inhibition of the growth of tumor tissues without significant adverse reactions	esophageal cancer	Chang et al. (2015)
	Co-encapsulating catalase (CAT) and acriflavine within the hydrophilic cavity of liposomes (ACF-CAT@Lipo)	Generates a significant amount of O_2_ both *in vitro* and *in vivo*, effectively alleviating tumor hypoxia, reduces the expression of hypoxia-related proteins, including HIF-1α, VEGF, and MMP-2, enhances the radiosensitivity of 125I brachytherapy	esophageal cancer	Yao et al. (2022)
Synergistic approach	graphdiyne anchored with CeO_2_ NPs	Significant advantage in mitigating tumor hypoxia, promote radiation-induced DNA damage inhibiting tumor growth and potentially reversing radioresistance	esophageal cancer	Zhou et al. (2021)
	Au NPs	Improves the radiosensitizing effect, intensify radiation-induced DNA damage, promote cell apoptosis, induce cell cycle arrest, and reduce colony-forming ability	esophageal cancer	Luan et al. (2022)

### 2.5 The applications of nanocarriers in immunity of esophageal cancer

The immune system, as an important component in the treatment of esophageal cancer, also possesses great research value. However, even though the nanodrugs have great application potential in immunity of esophageal cancer, the relevant references are still scarce. Utilizing immunostimulants activate immune cells of autosomatic immunity can effectively enhance the immune response and improve the anti-tumor ability within the body. In addition, the nanocarriers are appropriate as platform to long term and precisely deliver immunostimulants and the safety and efficacy of immunotherapy are improved while reducing side effects and complications of immune response. The immunosuppressive agents such as PD-1 can be delivered using nanodrugs, which can effectively suppress the immune escape of tumor cells, enhance the immune response in patients, and improve the efficacy of immunotherapy. Moreover, the standard first-line treatment for advanced esophageal cancer involves the use of immune checkpoint inhibitors in combination with chemotherapy. In the case of advanced esophageal and gastroesophageal junction cancer (including both squamous cell carcinoma and adenocarcinoma), the recommended first-line therapy includes combining pembrolizumab with platinum-based chemotherapy and fluoropyrimidine. For patients with advanced adenocarcinoma of the gastroesophageal junction, the recommended first-line treatment involves combining nivolumab with oxaliplatin and fluoropyrimidine. Lastly, for patients with advanced esophageal squamous cell carcinoma, the recommended first-line treatment is to combine camrelizumab with paclitaxel and platinum-based chemotherapy. Even though immune checkpoint inhibitor (ICI) is one of the most important tumor treatment methods, the application of nanodrugs in the immunotherapy of esophageal cancer is still limited, and immune nano-drugs delivery system can improve the therapeutic effect of immune checkpoint inhibitors on tumors (Qian et al., 2022). So, a large number of clinical trials are still necessary to verify the role of nanodrugs in the immunotherapy of esophageal cancer.

### 2.6 The applications of nanocarriers in esophageal cancer surgery

Local recurrence and lymph node metastasis are crucial factors affecting the survival rate of patients with esophageal cancer. However, in patients who experience recurrence after curative surgery for esophageal cancer, the majority present with lymph node metastasis, with a remarkably high proportion of 44.5% (Wang et al., 2010). Currently, in China, the main surgical approaches for esophageal cancer involve two routes: via left thoracic and via right thoracic. Nevertheless, it has been revealed that the left thoracic approach is associated with a high incidence of lymph node metastasis in the upper mediastinum and supraclavicular region, ranging from 30% to 40%. In contrast, via the right thoracic approach, encompassing a complete lymph node dissection of the chest and/or neck, can significantly reduce the rate of lymph node metastasis and recurrence. However, despite the complete lymph node clearance achieved with this approach, it is accompanied by prolonged surgical time, increased trauma, and a higher likelihood of irreversible and fatal complications, without necessarily improving the quality of life and survival benefits for some patients. Hence, it is of utmost clinical importance to selectively remove suspicious positive metastatic lymph nodes in patients with esophageal cancer, avoiding excessive lymph node dissection.

Nanocarbon lymph node tracers represent a novel type of lymphatic tissue-active dye designed for identifying sentinel lymph nodes, observing lymphatic drainage patterns, and guiding curative surgery (Wang C. et al., 2020; Lin et al., 2020). Meanwhile, the nanocarbon lymph node tracers possessing high affinity for lymph nodes and carbon adsorption capacity can effectively adsorb specific chemotherapeutic drugs, facilitate lymphatic-targeted preoperative or intraoperative chemotherapy, and ultimately enhance the quality of life for individuals with tumors. In the context of endoscopic esophageal cancer curative surgery, the use of nanocarbon tracers has been found to increase the number of dissected lymph nodes and detect more metastatic lymph nodes, resulting in reduced postoperative bleeding, drainage, and recurrent laryngeal nerve injury compared to the control group without nanocarbon tracers (Liu P. et al., 2020). Since chemotherapy drugs face challenges in reaching lymph nodes through the bloodstream, the efficacy of systemic chemotherapy for metastatic lymph nodes is limited, resulting in insufficient improvement in survival rates for some patients even if they undergo surgery. Consequently, targeted preoperative or intraoperative chemotherapy for lymph node metastasis assumes paramount significance. By leveraging the characteristics of large molecular substances and particles that are easily engulfed by the lymphatic system, preoperative or intraoperative lymphatic-targeted chemotherapy utilizing drug-loaded nanoparticles enhances the drug concentration within local lymph nodes, prolongs drug action duration, and mitigates drug toxicity and side effects. Although the application of nanocarbon in esophageal cancer surgery is currently limited, the aforementioned studies collectively demonstrate that nanocarbon improves the quality of life and prolongs survival time for patients undergoing curative surgery for esophageal cancer, underscoring its immense value in this therapeutic context.

Endoscopic submucosal dissection (ESD), is an advanced minimally invasive technique using various electrosurgical knives and endoscopy for the meticulous submucosal dissection of the *en bloc* resection of lesions larger than 2 cm. ESD offers several advantages over traditional surgical approaches, including accurate pathological staging, shorter operation time, faster recovery, shorter hospital stay, and reduced medical costs (Kantsevoy et al., 2008). Even though ESD has become the preferred treatment modality for early-stage gastrointestinal cancers and precancerous lesions, the major challenges associated with ESD are the higher risk of bleeding and perforation (Yamamoto, 2012). To mitigate these complications, injecting appropriate submucosal injection media, a thick liquid or gas cushion, allows for effective and prolonged separation between the lesion and muscular layer playing a critical role, which is crucial for successful ESD. Traditional submucosal injection media, such as physiological saline, are quickly absorbed by the surrounding tissue, necessitating repeated injections during the ESD procedure (Zhou et al., 2014), while hypertonic solutions can cause tissue damage (Fujishiro et al., 2005; Katsinelos et al., 2008). In contrast, hyaluronic acid (nano-)hydrogel has many advantages such as maintaining a favorable submucosal fluid cushion and excellent safety, making it the preferred choice for submucosal injection during ESD (Jung and Park, 2013). Similarly, hydroxyethyl cellulose, a synthetic product, can trigger antigen-antibody reactions (Lenz et al., 2010), while fibrinogen mixture carries the risk of viral transmission, including hepatitis (Uraoka et al., 2009). Recent advancements in submucosal injection media have introduced novel options. For example, carbon dioxide gas has shown promising results as a submucosal buffering agent, significantly extending the elevation time of the submucosal layer compared to physiological saline (Uraoka et al., 2011). Sodium alginate (nano-) hydrogel has emerged as a potential alternative to hyaluronic acid, exhibiting favorable characteristics as a submucosal injection medium (Kang et al., 2013). Additionally, elastic polymer submucosal layer elevation has demonstrated superior performance over physiological saline, providing greater elevation height and promoting effective post-ESD mucosal healing (Tran et al., 2012). Moreover, a new approach involves using DMEM/F12 cross-linked chitosan hydrogel, which can be transformed into an insoluble hydrogel through brief exposure to ultraviolet irradiation. Compared to hypertonic saline and hyaluronic acid, this hydrogel exhibits sustained elevation and clear margins, enabling precise endoscopic submucosal dissection without complications such as bleeding or perforation. However, because of the longer degradation time its prolonged degradation time *in vivo* may delay wound healing (Kumano et al., 2012). In summary, ESD has revolutionized the treatment of early-stage gastrointestinal cancers and precancerous lesions. To address the challenges of bleeding and perforation, the selection of suitable submucosal injection media is crucial. While traditional options have limitations, recent advancements, such as (nano-) hydrogel hyaluronic acid, sodium alginate, cross-linked chitosan hydrogel and carbon dioxide gas, offer promising alternatives with improved safety and efficacy. Continued research in this field is essential to optimize the outcomes of ESD and further enhance patient care.

### 2.7 The applications of nanocarriers in new treatment of esophageal cancer

Nowadays, there are two common new methods used for non-surgical tumor therapy, i.e., photothermal therapy (PTT) and photodynamic therapy (PDT). In PTT, tumor cells are eliminated by subjecting a photosensitizer to light of a specific wavelength, which induces localized heating and subsequent cell death, and as for PDT, a photosensitizer that, upon exposure to specific light, generates a high concentration of reactive oxygen species (ROS) leading to tumor cell eradication. While the use of photosensitizers is essential in PDT, PTT offers the advantage of enhanced therapeutic efficacy and outcomes without the need for exogenous heat-generating agents.

#### 2.7.1 The PTT nanocarriers in esophageal cancer

Tumor hyperthermia therapy employs various techniques such as ultrasound, microwave, radiofrequency, and magnetic field to heat tumor tissue. This treatment approach demonstrates a higher specificity towards tumors due to their inherent heat tolerance, but the therapy efficacy is limited because the damage to adjacent healthy tissues should be minimized (Gonçalves et al., 2020). Photothermal therapy (PTT) has emerged as a promising solution to overcome this challenge by utilizing photothermal agents to precisely heat the targeted area and restrict thermal damage to the tumor tissue. However, the effectiveness of PTT relies on improved light absorption and enhanced photothermal conversion efficiency (Wang et al., 2020b). Noble metal nanoparticles, commonly employed as photothermal agents for *in vivo* therapy, offer a less invasive experimental technique that ensures effective cancer treatment (Huang et al., 2006). The efficacy of PTT depends on two primary factors: i) the light source, such as lasers, which enable deeper tissue penetration by emitting light in the spectral range of 650–900 nm, and ii) light-absorbing nanoparticles that facilitate photothermal ablation through efficient conversion of light into heat within picosecond timescales (Chen et al., 2007). These nanoparticles exhibit strong absorption in the near-infrared (NIR) region of the electromagnetic spectrum, particularly in the range of 650–900 nm, due to their surface plasmon resonance (SPR) effect, and notably, the absorption coefficient of nanoparticles is 4–5 times higher than that of photothermal dyes owing to their SPR characteristics (Jain et al., 2008). Spherical gold nanoparticles typically exhibit their maximum SPR absorption peak in the visible spectrum, around 520 nm. Huang et al. identified gold nanorods as effective photothermal agents due to their longitudinal absorption bands in the NIR region resulting from SPR oscillation (Huang et al., 2010). Specifically, small-diameter gold nanorods are preferred as NIR photothermal converters for *in vivo* applications due to their high absorption cross-section beyond the tissue absorption spectrum. Moreover, the nanorods hold promise as an ablative component for cancer treatment, as it enables NIR light transmission through human skin and tissue (Kuo et al., 2010). Another approach involves utilizing silica-gold with core-shell structure to enhance the photothermal effect within cancer cells because of the controlled thermotherapy generated under laser (Terentyuk et al., 2009). A research by Chen et al. explored a gold nanoshell-based system for cancer targeting and PTT in HER2-overexpressing and drug-resistant ovarian cancer cells (OVCAR3). This nanomaterial was designed to facilitate simultaneous fluorescence optical imaging and magnetic resonance imaging, and the study revealed selective killing of OVCAR3 cells upon NIR laser irradiation using the nanocomposite system (Chen et al., 2010). Additionally, Arnfield et al. conducted an observational clinical trial involving core-shelled gold-silica induced hyperthermia, followed by NIR light exposure, in patients with squamous cell carcinoma of the head and neck (Arnfield et al., 1992). In another study, McGrath et al. conducted research on the synthesis and efficacy of palladium-gold nanocomposites for enhanced photothermal therapy (PTT) applications (McGrath et al., 2015). They successfully demonstrated the potential of PTT by combining these nanocomposites with 808 nm diode laser radiation, leading to the effective eradication of HeLa cells *in vitro* and the destruction of cervical cancer cells in HeLa tumor xenografts in male B9 mice. Similarly, Shen et al. reported the effects of photothermal ablation mediated by magnetic nanoparticle clusters on both *in vitro* and *in vivo* cancer models (Shen et al., 2015). Their findings revealed that clustered Fe_3_O_4_ nanoparticles exhibited significantly enhanced NIR absorption compared to magnetic Fe_3_O_4_ nanoparticles alone, and the clustered nanoparticles achieved higher cellular toxicity against A549 cells by generating elevated temperatures after NIR irradiation at 808 nm. Furthermore, an *in vivo* study of photothermal therapy in a tumor-bearing mouse model (A549) also demonstrated that clustered Fe_3_O_4_ nanoparticles had superior therapeutic efficacy compared to individual free Fe_3_O_4_ nanoparticles (Alshehri et al., 2020). As well as Fe_3_O_4_ nanoparticles, the dispersive gold nanoparticles have no damage to tissues while once they aggerate and irradiated by near-infrared (NIR) light, malignant tissues can be selectively destroyed. Yan Li et al. investigated the feasibility of using chitosan-coated gold/gold sulfide (CS-GGS) nanoparticles for photothermal ablation therapy of esophageal adenocarcinoma. In their study, a rat model of esophagojejunostomy was employed for *in vivo* ablation experiments, and three human esophageal cell lines were utilized to assess the response of cancerous and benign cells to NIR light following treatment with CS-GGS nanoparticles. The results demonstrated that both cancer tissue and cancer cells exhibited higher uptake of gold nanoparticles, resulting in complete ablation upon exposure to NIR light. In contrast, benign tissue and non-cancerous cells exhibited lower nanoparticle uptake and maintained viability after NIR light exposure. Therefore, CS-GGS nanoparticles represent a promising intracavitary treatment option for NIR light-mediated ablation of esophageal cancer (Li et al., 2013). Moreover, the silica nanostructures were effectively covered onto the synthesized Cu_9_S_5_ to form Cu_9_S_5_@MS core-shell nanostructures. This nano-system successfully utilized for photothermal removal of esophageal squamous carcinoma cells and NIR therapy with good biocompatibility (Wang et al., 2019).

#### 2.7.2 The PDT nanocarriers in esophageal cancer

Photodynamic therapy (PDT) is gaining attention as a promising treatment modality for oral diseases due to its numerous advantages, and this physical therapy has now evolved into a viable treatment option within the realm of cancer chemotherapy. PDT has been explored as a treatment approach for various cancer types, including skin cancer, head and neck cancer, esophageal cancer, gastric cancer, pancreatic cancer, bladder cancer, prostate cancer, and lung cancer. PDT involves the localized application or systemic administration of a photosensitizer, which accumulates in the target tissue and undergoes a light-induced photochemical reaction, resulting in irreversible tissue damage or necrosis. PDT exploits the ability of photosensitizers to absorb specific wavelengths of light, generating oxygen-based molecular species that induce cytotoxic effects, and these reactive species cause damage to cellular membranes and subcellular organelles, ultimately leading to cell death through apoptosis, necrosis, or autophagy. The effectiveness of PDT relies on the photosensitizers’ capacity to selectively produce singlet oxygen at therapeutic concentrations within the tumor site (Chen et al., 2020). A wide range of organic photosensitizers, including porphyrins, dipyrrolemethene or phthalocyanine derivatives, curcumin, etc., have been extensively investigated for PDT in clinical and preclinical settings. To enhance the *in vivo* therapeutic efficacy of nanoparticle-mediated PDT in cancer, these photosensitizers are often encapsulated within nanocarriers.

Furthermore, in recent years, nanomaterials with inherent photodynamic properties, such as graphene, quantum dots (QDs), and titanium dioxide (TiO_2_) nanoparticles, have emerged as potential alternatives to overcome the limitations associated with hydrophobic photosensitizers (Sivasubramanian et al., 2019). Unterweger et al. conducted a study to develop iron oxide nanoparticles loaded with curcumin as carriers for photodynamic therapy (PDT) (Unterweger et al., 2015). Flow cytometry analysis of Jurkat human T-cell leukemia cell line demonstrated that the pure nanoparticle system and curcumin alone did not exhibit toxicity when cells were not exposed to light. However, when curcumin was delivered in combination with the nanoparticles and light irradiation was applied, concentration- and time-dependent cancer cell death was induced due to the formation of reactive oxygen species. In another study, Li et al. developed hybrid nanoparticles consisting of thiolated heparin-deprotonated chlorophyll A (PhA) conjugated iron oxide and gold nanoparticles (Fe_3_O_4_/Au-NP) for efficient monitoring of PDT (Li L. et al., 2014). Experimental results showed that A549 cancer cells treated with the hybrid nanoparticles and subjected to light irradiation displayed significant phototoxicity and strong fluorescence signals. Furthermore, Shen et al. designed a novel nanocomposite for tumor targeting, composed of quantum dot-zinc-porphyrin nanocomplexes encapsulated in folate-modified phospholipid polymers (Shen et al., 2017). This system exhibited a high payload of porphyrin, leading to a significantly increased production of singlet oxygen, and *in vivo* studies demonstrated the preferential accumulation of the developed nanoparticle system in tumor tissue, allowing for effective monitoring of PDT using non-invasive fluorescence imaging techniques in a mouse model. Additionally, Murakami et al. investigated the ability of semiconductor-rich and metal-rich single-walled carbon nanotubes to generate reactive oxygen species under near-infrared light (808 nm) irradiation (Murakami et al., 2012). Their findings indicated that the semiconductor-rich single-walled carbon nanotubes exhibited a stronger photodynamic effect compared to the metal-rich counterparts. The representative nanomaterials for new treatment of esophageal cancer were summarized in Table 6.

**TABLE 6:** Representative nanomaterials for new therapy method of esophageal cancer.

Types	Materials	Features and findings	Application	References
PTT	Gold nanoshell	facilitate simultaneous fluorescence optical imaging and magnetic resonance imaging, selectively kill of OVCAR3 cells upon NIR laser irradiation	Ovarian cancer cells	Chen et al. (2010)
	Palladium-gold nanocomposites	Lead to the effective eradication of HeLa cells *in vitro* and the destruction of cervical cancer cells in HeLa tumor xenografts in male B9 mice	Cervical cancer	McGrath et al. (2015)
	Clustered Fe_3_O_4_ NPs	enhance NIR absorption and achieve higher cellular toxicity against A549 cells by generating elevated temperatures after NIR irradiation at 808 nm	Lung cancer	Alshehri et al. (2020)
	Chitosan-coated gold/gold sulfide NPs	Complete ablation upon exposure to NIR light	Esophageal cancer	Li et al. (2013)
PDT	Iron oxide NPs loaded with curcumin	Generate reactive oxygen species after light irradiation and induce concentration- and time-dependent cancer cell death	T-cell leukemia	Unterweger et al. (2015)
	Thiolated heparin-deprotonated chlorophyll A (PhA) conjugated Fe_3_O_4_/Au-NPs	A549 cancer cells treated with the hybrid nanoparticles and subjected to light irradiation display significant phototoxicity and strong fluorescence signals	Lung cancer	Li et al. (2014a)
	Quantum dot-zinc-porphyrin nanocomplexes encapsulated in folate-modified phospholipid polymers	High payload of porphyrin, lead to a significantly increased production of singlet oxygen, and accumulate the developed nanoparticle system in tumor tissue, allow for effective monitoring of PDT using non-invasive fluorescence imaging techniques in a mouse model		Shen et al. (2017)

## 3 The applications of nanocarriers in complications of esophageal cancer

### 3.1 Esophageal stenosis or esophageal obstruction

Dysphagia, a prominent symptom in the majority of advanced esophageal cancer patients who have eliminated the occasion of curative surgical interventions. Although the insertion of esophageal stents can alleviate eating difficulties to some extent, it fails to address tumor control. To explore new treatment avenues for late-stage esophageal cancer patients, researchers have incorporated nanoparticles into stents coated with a fibrous membrane. Subsequently, through intracavity photodynamic therapy, they observed a substantial increase in animal survival time, presenting a novel therapeutic direction. Zhao et al. reported that a patient with esophageal squamous cell carcinoma experienced local progression following chemoradiotherapy, resulting in nutritional deficiencies and mild anemia due to impaired eating. Facilitated by innovative localization techniques, i.e., X-ray fluoroscopy and albumin-bound paclitaxel, photodynamic therapy was employed to this patient, leading to positive clinical outcomes (Zhao et al., 2022). Li et al. explored the application of polydopamine (PDA) and polyethyleneimine (PEI) coatings on 317L stainless steel (317LSS) esophageal stents, and after modification the surface exhibited tumor growth inhibition, endowing the stents with sustained anticancer functionality, thus offering an ideal strategy for mitigating esophageal restenosis (Zhang et al., 2017). Similarly, Bai et al. coated the surface of 317LSS esophageal stents with a layer composed of polydopamine/polyethyleneimine/5-fluorouracil (PDA/PEI/5-Fu), demonstrating strong antitumor and anti-restenosis properties (Bai et al., 2018). In a related study, Zhang et al. also found that the PDA/PEI/5-Fu composite layer on the esophageal stents inhibited the viability of pathological cells and the expression of E-cadherin, thus blocking the NF-κB signaling pathway. Additionally, the loaded 5-Fu suppressed the release of inflammatory factors (TNF-α and IL-1β), promoted macrophages to release anti-inflammatory/anti-tumor factors (IL-10 and IL-4), and inhibited the migration of pathological cells. Both PEI and 5-Fu contributed to the upregulation of Bax and Caspase-3 (pro-apoptotic factors) and the downregulation of Bcl-2 (anti-apoptotic factor) in esophageal tumor cells. These comprehensive findings suggest that the PDA/PEI/5-Fu coating holds significant potential for multi-pathway anticancer and anti-inflammatory effects in the surface modification of esophageal stents (Zhang et al., 2020). Song and co-workers developed an innovative approach involving the application of a highly efficient gold nanoshell (AuNS) coating on stents, and in brief, the surface of the stents was coated with polydopamine to serve as an anchor and template for Au^3+^ ions. The resulting AuNS-coated stents exhibited enhanced temperature elevation upon near-infrared (NIR) laser irradiation in pork and pig intestines. Compared to bare metal stents, these AuNS-modified stents hold immense potential for clinical implementation, as they can facilitate duct channel opening and impede tumor growth (Song et al., 2016).

Cho et al. developed a self-expanding metal stent coated with gold nanoparticles for photothermal therapy under near-infrared laser irradiation, which successfully treated granulation tissue formation after stent placement in the rat esophagus (Cho et al., 2021). In another study, Liu et al. designed a biodegradable composite scaffold by coating poly (lactic-co-glycolic acid) (PLGA) with paclitaxel (PTX) onto a magnesium-based woven scaffold. Compared to scaffolds without PTX-PLGA coating, the PTX-PLGA scaffolds exhibited higher radial force and faster degradation in an acidic environment, effectively promoting fibroblast apoptosis *in vitro* (Liu et al., 2022).

### 3.2 Oesophagitis

The clinical results show that during the radiotherapy process, there is a risk of oesophagitis which is an adverse concomitant disease hindering therapy. Thus, inhibiting concomitant oesophagitis becomes a research focus. Michael W. et al. administered intragastric injections of manganese superoxide dismutase (MnSOD)-plasmid/liposome complexes into experimented C3H/HeNsd mice. The results showed that this treatment effectively prevented radiation-induced esophagitis (Epperly et al., 2001). Additionally, Niu et al. investigated the effects of MnSOD-plasmid/liposome complexes in mice and found that they improved esophageal radiation tolerance by enhancing the engraftment and self-renewal of bone marrow-derived progenitor cells in the esophageal squamous epithelium (Niu et al., 2008). Furthermore, in an *in vitro* study, Michael W. et al. also transfected human esophageal segments with the MnSOD-plasmid/liposome complexes, demonstrating their ability to prevent cell apoptosis induced by ionizing radiation and alleviate radiation-induced esophagitis (Epperly et al., 2000).

## 4 The exploration of nanocarriers in Chinese patent medicine

The rapid development of nanomaterials provides excellent carriers for Chinese patent medicine which is also widely used to treat cancer including esophageal cancer, and the exploration of nanocarriers used in Chinese patent medicine has never stopped. For instance, Hu et al. prepared a nanocomposite material comprising chitosan-sodium alginate-polyethylene glycol-crocin which can inhibit the growth of KYSE-150 esophageal cancer cells by effectively increasing the generation of reactive oxygen species (ROS) and inducing apoptotic cell death (Hu et al., 2022). Moreover, Al-Hazmi et al. purified cyanine dye from *Pseudomonas aeruginosa* and immobilized it onto nanochitosan. The anticancer activity of dissociative cyanine dye and nanocyanine dye was evaluated against CLS-145 gastric cancer cells, AsPC-1 pancreatic cancer cells, HCT116 colon cancer cells, KYSE-410 esophageal cancer cells, and HepG2 liver cancer cells, and the antibacterial activity of these dyes against bacteria associated with gastrointestinal cancer biopsies, including *Helicobacter pylori*, *Fusobacterium* nucleatum, enterohemorrhagic *Escherichia coli*, *Clostridium difficile*, and Porphyromonas gingivalis, was also examined. The results demonstrated notable anticancer and antibacterial effects for both dissociative cyanine dye and nanocyanine dye, and the nanocyanine dye exhibiting superior activity in suppressing cancer growth and combating bacterial strains (Al-Hazmi and Naguib, 2022). Furthermore, Li et al. synthesized two novel polymer-drug conjugates, docetaxel-polyvinyl alcohol and docetaxel-chitosan, via alcoholysis reactions. These nanocomposites exhibited dose-dependent inhibition of ECA-109 human esophageal cancer cells and EMT6 mouse mammary cancer cells and they induced apoptosis in ECA-109 cells and caused cell cycle arrest at the S phase. Notably, the conjugates demonstrated significant antitumor activity in an EMT6 tumor-bearing mouse model, surpassing the tumor suppression rates of free docetaxel (Li M. et al., 2014). Martin et al. investigated the synthesis and characterization of a novel nanosystem comprising gold nanorods loaded with curcumin and then incorporating them into polymer nanoparticles (Martin et al., 2015). The viability of adenocarcinoma cells was significantly reduced because both gold nanorods and curcumin exhibited effective photothermal effects. Therefore, the combination of gold nanorods and curcumin presents a safe and effective approach for eradicating precancerous esophageal adenocarcinoma (Li et al., 2022). Zhan encapsulated β-elemene within the pores of mesoporous silica nanoparticles, while artemisinin was electrostatically adsorbed onto the nanoparticle surface, resulting in a synergistic dual-drug nanosystem. This nanosystem demonstrated excellent antitumor effects both *in vitro* and *in vivo*, offering a promising therapeutic option for esophageal cancer treatment (Zhan et al., 2020). Zou et al. employed a simple biosynthetic method to prepare copper oxide nanoparticles (CuO nanoparticles) using an extract from Astragalus membranaceus at ambient temperature. These nanoparticles exhibited cytotoxicity against the human esophageal cancer cell line KYSE30, while no significant cytotoxicity was observed in human dermal fibroblasts (Zou et al., 2020). Zhang et al. successfully synthesized nickel nanoparticles using the aqueous extract of Calendula officinalis leaves in a water-based medium, which demonstrated significant inhibitory and antioxidant effects against esophageal cancer cell lines (Zhang et al., 2021). Jiang et al. synthesized biogenic zinc oxide nanoparticles (ZnO nanoparticles) using a plant extract obtained from Arisaema heterophyllum showing good biocompatibility and exhibited anti-esophageal cancer activity (Jiang et al., 2020). Cyclin D1 is commonly overexpressed in esophageal squamous cell carcinoma and plays a crucial role in tumor development and proliferation (Dey et al., 2015). Curcumin possesses anti-inflammatory, antioxidant, and anticancer properties and has been shown to have therapeutic effects against esophageal cancer. The bioavailability of curcumin in free form is limited, but in contrast, nanocurcumin significantly reduced the proliferation of esophageal squamous cell carcinoma KYSE-30 cells without affecting the proliferation of normal esophageal cell lines. Furthermore, it downregulated the expression of Cyclin D1 (Hosseini et al., 2018).

Pi et al. devised a strategy to enhance the anticancer efficacy by targeting cancer cells with overexpressed epidermal growth factor receptor (EGFR) using GE11 peptide-conjugated selenium nanoparticles for the delivery of colchicine (Pi et al., 2017). This study revealed that this method achieved efficient loading of GE11 peptide-conjugated selenium nanoparticles, resulting in enhanced uptake by tumor cells and improved tumor suppression while reducing nonspecific toxicity. The nanoparticles exhibited preferential accumulation in lysosomes, enabling the release of colchicine in acidic conditions. Subsequently, upon translocation to the cytoplasm, they induced disruption of membrane integrity (Li et al., 2022).

## 5 The toxicity of inorganic nanocarriers

Even though there are extensive preclinical studies conducted over the past two decades have demonstrated the distinct diagnostic and therapeutic opportunities offered by inorganic and metallic nanoparticles because of their unique physicochemical properties, setting them apart from polymer and lipid nanoparticles. These inorganic nanoparticles are well-positioned to address several challenges that remain unresolved in clinical applications. However, the issue of toxicity has overshadowed their potential therapeutic and diagnostic benefits, warranting careful consideration. In general, nanomaterials tend to accumulate within various cell types, including macrophage-like cells such as tissue and blood phagocytes, as well as reticuloendothelial system (RES) cells, and their deposition has been observed in tissues such as lymph nodes, bone marrow, brain, spleen, adrenal glands, liver, and kidneys, highlighting their widespread distribution. The impact of physicochemical properties of inorganic nanoparticles, including size, shape, solubility, surface charge, chemical structure, reactivity, and surface modifications, has been extensively investigated and is well-documented (Mohammadpour et al., 2019), and their safety concerns associated with nanomedicines are well-documented, particularly regarding DNA damage and oxidative stress (Damasco et al., 2020). In this discussion, we focus on the biocompatibility issues pertaining to commonly used and promising inorganic nanoparticles (NPs). Taking gold nanoparticles (AuNPs) as an illustrative example, it has been observed that colloidal AuNPs with sizes ranging from 10 to 50 nm may exhibit greater toxicity compared to larger particles measuring 100–200 nm. Specifically speaking, AuNPs within the size range of 2.8–38 nm have been found to induce heightened toxicity and immune responses (Yen et al., 2009). Conversely, there is an exception that AuNPs measuring 15 nm demonstrate no toxicity to cells, even at concentrations 60 times higher than the IC50 of smaller AuNPs (Dauty and Verkman, 2005). These findings underscore the size-dependent toxicity of AuNPs. Specifically, smaller AuNPs, especially those below 15 nm, have been found to significantly upregulate pro-inflammatory genes such as interleukin-1, interleukin-6, and tumor necrosis factor-α, resulting in a reduction in macrophage population (Adewale et al., 2019). The relationship between AuNP shape and toxicity has also been investigated, with a study conducted by Sun et al. revealing shape-dependent *in vivo* toxicity of AuNPs, i.e., rod-shaped AuNPs demonstrated the highest toxicity, followed by cubic AuNPs, while spherical AuNPs exhibited the best biocompatibility. (Sun et al., 2011). Additionally, the study highlighted the preferential accumulation of all AuNPs in the liver and spleen, and consistent with the biodistribution patterns of other nanomedicines, AuNPs, following intestinal absorption, exhibit high distribution in the blood, brain, lungs, heart, kidneys, liver, and spleen (Qiu et al., 2010). Moreover, positively charged spherical AuNPs exhibited greater toxicity compared to negatively charged particles of the same size.

Moving on to other widely explored inorganic nanoparticles with significant safety concerns, Quantum Dots (QDs) have been extensively investigated for their diagnostic and therapeutic potential. However, it is known that QDs composed of heavy metal ions such as Cd^2+^, released from QDs lacking polymer protection when exposed to ultraviolet radiation, can induce lung and kidney damage (Schmid et al., 2017). *In vitro* studies on CdSe QDs have shown acute toxicity to primary liver cells, attributed to the release of free Cd^2+^ ions (Kargozar et al., 2020). Among the metal-based nanoparticles, Superparamagnetic Iron Oxide Nanoparticles (SPIONs) have garnered considerable attention due to their biodegradability and relatively lower toxicity under *in vivo* conditions (Lee et al., 2010). SPIONs have been extensively evaluated and developed for diagnostic applications, and more recently, they have shown promise in the field of magnetic hyperthermia therapy. However, some iron oxide materials have been withdrawn from use due to toxicity concerns or a lack of clinical benefits. Toxicity levels of SPIONs have been a subject of debate among researchers, primarily based on viability test results, and the biodistribution studies have shown the accumulation of SPIONs in various tissues and organs, including the brain. Nevertheless, a clear understanding of acute toxicity, genetic toxicity, immune toxicity, reproductive toxicity, and neurotoxicity of SPIONs is still lacking, and results vary across different animal models. Lastly, gadolinium (Gd), commonly used as a contrast agent in clinical settings, including cancer treatment, deserves discussion. Despite its widespread use, gadolinium nanoparticles have raised concerns regarding human toxicity, and patients with pre-existing renal impairment may develop Nephrogenic Systemic Fibrosis (NSF), a condition characterized by systemic tissue fibrosis, following the administration of gadolinium (Alshehri et al., 2020). It is crucial to further investigate the toxicity profile of gadolinium nanoparticles, considering their potential applications in theragnostic.

## 6 Challenges for clinical translational and approval of cancer nanomedicine

The process of translating cancer nanomedicines from laboratory research to clinical applications and commercialization is a time-consuming and resource-intensive endeavor. While a limited number of nanomedicines, including Doxil^®^, Myocet^®^, Abraxane^®^, Depocyt^®^, and Genexol^®^, have successfully obtained regulatory approval for use in chemotherapy, the overall number remains relatively low. This phenomenon highlights the significant disparity that exists between the laboratory-level development of nanomedicines and their subsequent translation into clinical practice and large-scale industrial production (Ferrari, 2005).

The following paragraphs aim to address the critical issues that impede the clinical development and regulatory approval of cancer nanomedicines: 1) One major and pivotal drawback lies in our limited understanding of the pathological and physiological complexities and heterogeneity of tumor sites that influence patient selection. Identifying which patients are most likely to benefit from nanoparticle-based chemotherapy remains a challenge (Maeda, 2015). 2) Additionally, our knowledge of the behavior of nanoparticles inside the body is predominantly based on animal data, and the animal models used often fail to accurately replicate the true *in vivo* conditions. 3) Typically, systemically administered nanoparticles accumulate in solid tumors via the enhanced permeability and retention (EPR) effect, but several crucial aspects related to the interpretation of EPR have been significantly overlooked, including nanoparticle-protein interactions, blood circulation, tumor tissue penetration, and cellular internalization. Furthermore, the behavior of nanoparticles *in vivo* is greatly influenced by their characteristics, such as size, geometry, surface features, and consequently, there are numerous factors governing the EPR-driven *in vivo* behavior of nanoparticles that cannot be reliably predicted solely from animal data. In summary, these challenges underscore the complex nature of translating nanomedicines into clinical applications. To overcome these hurdles, a more profound understanding of tumor heterogeneity, as well as the impact of nanoparticle properties on their *in vivo* behavior, is imperative. This necessitates further research and exploration to bridge the gap between preclinical studies and clinical translation.

In addition, one of the key obstacles lies in optimizing the physicochemical parameters necessary for the successful development of therapeutic nanoparticles. This optimization process is essential but presents a significant barrier to the rapid and reproducible synthesis of large quantities of nanoparticles with distinct characteristics due to the involvement of multiple steps or intricate techniques. To overcome this challenge, new scaling strategies and alternative approaches to formulation development must be explored (Karnik et al., 2008). Additionally, the clinical development of nanomedicines faces ambiguity in the requirements of chemistry, manufacturing, and controls (CMC) and good manufacturing practice (GMP). Scaling up the production of more complex nanomedicines introduces challenges in maintaining compliance with CMC and GMP standards. This necessitates improvisation and adaptation of existing manufacturing processes to meet regulatory requirements (Rolland et al., 2005). To date, the evaluation of the enhanced permeability and retention (EPR) effect and nanoparticle penetration in human tumor metastasis models has proven to be of significant importance, considering the impact of tumor metastasis on cancer-related mortality (Zuckerman et al., 2014). The successful clinical translation of cancer nanomedicines heavily relies on the introduction of animal models that faithfully replicate the heterogeneity and anatomical histology observed in human tumors (Meads et al., 2009).

**FIGURE 4:**
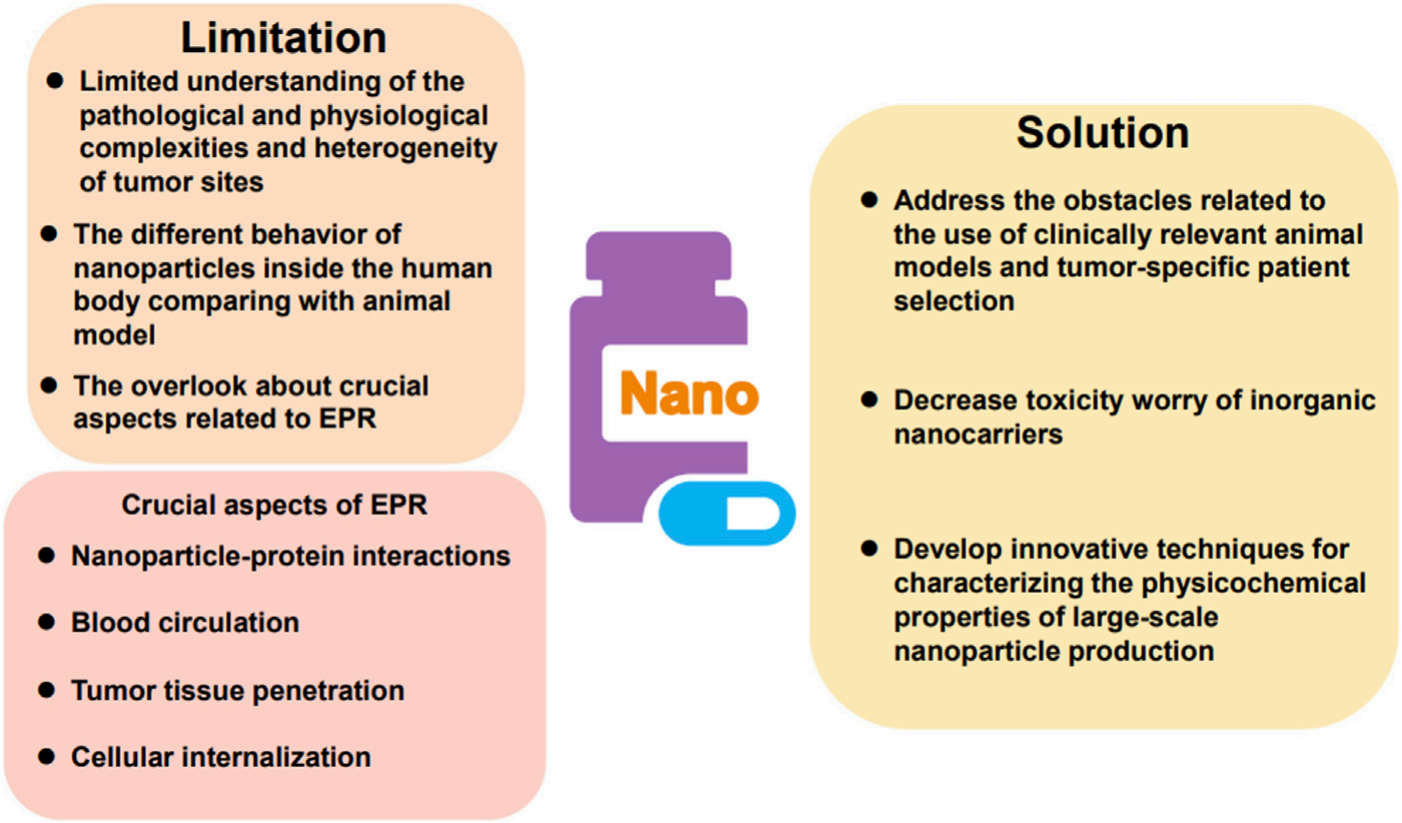
The limitation and solution of nanomaterials for therapy and diagnosis of esophageal cancer.

In summary, addressing the obstacles related to the use of clinically relevant animal models, tumor-specific patient selection, decrease toxicity worry of inorganic nanocarriers and the development of innovative techniques for characterizing the physicochemical properties of large-scale nanoparticle production are crucial steps towards the successful clinical translation of cancer nanomedicines (Figure 4).

## 7 Discussion and outlook

The field of cancer nanomedicine encompasses two primary areas: cancer nanotherapy and nanodiagnostics. Over the past four decades, significant advancements have been made in cancer treatment through the development of cancer nanomedicines including extending overall survival, improving quality of life, and reducing toxicity. The approval of liposomal doxorubicin (Doxil^®^) as the first cancer nanomedicine two decades ago marked a turning point, leading to substantial investment and research in this field by both industry and academia. However, translating the promising preclinical results of cancer nanomedicines into clinical success has proven to be a challenging task. Despite these challenges, there have been recent approvals of nanomedicine products for the treatment of orphan cancers, as well as ongoing developments in cancer nanomedicines for cancer immunotherapy, while the approval of lipid nanoparticle-formulated mRNA as a vaccine for COVID-19 in late December 2020 has also reignited interest in cancer nanomedicines and renewed hopes for the development of non-viral cancer vaccines. The field of cancer nanomedicine has garnered more interest and investment compared to any other therapeutic area reflected in the global revenue trends and forecasts in the nanomedicine market since 2016, where the market share of cancer nanomedicine is significantly higher than that of other therapeutic areas. Currently, there are 18 approved cancer nanomedicines, many of which have shown positive results in Phase III trials holding great promise for improving cancer treatment. Additionally, the successful approval of nanoanalogue for several cancer nanomedicines is helping to alleviate the high financial burden associated with cancer treatment. It is anticipated that the use of generic drugs will further enhance affordability and accessibility of nanomedicines in the near future. In conclusion, the field of cancer nanomedicine has made significant progress in improving cancer treatment, and despite the challenges faced in translating preclinical success to clinical efficacy, there is a continued drive for research and development in this field. The potential of nanomedicines to revolutionize cancer therapy and their growing market presence highlight the importance of ongoing investment and exploration in cancer nanomedicine.

The field of nanotheranostics and nanodiagnosis are rapidly evolving research areas that has not yet reached clinical standards. While some nanodiagnostic systems exhibit significant diagnostic efficacy, they often lack therapeutic capabilities, and on the other hand, certain systems have demonstrated primary therapeutic indicators but limited imaging capabilities. To overcome these limitations, extensive efforts have been made to explore various nanomaterials and modification techniques, assessing their *in vivo* performance and paving the way for potential clinical trials. Currently, most research in nanodiagnostics focuses on evaluating their diagnostic and therapeutic applications using animal models, yielding promising results. However, the translation of these systems to human subjects has proven challenging due to differences in the diffusion mechanisms between animals and humans. Moreover, the potential toxicity and safety concerns associated with nanotheranostics when used in humans have been a significant area of investigation, such as nanodiagnostic systems based on carbon nanotubes and metal nanoparticles have raised safety concerns due to their slow degradation and potential *in vivo* accumulation. In response, researchers have explored strategies such as surface coating with biocompatible/biodegradable polymers or synthesizing nanodiagnostic systems using clinically approved nanomaterials to enhance their *in vivo* efficacy and address safety concerns. While substantial progress has been made in designing complex nanotheranostic systems worldwide, significant efforts are still needed before their widespread clinical application becomes a reality. Nevertheless, the future of nanotheranostics in clinical practice is within reach, and with continued dedication and research, further advancements can be expected.
